# Update of Residential Tetrachloroethylene Exposure and Decreases in Visual Contrast Sensitivity

**DOI:** 10.1289/ehp.112-a862c

**Published:** 2004-11

**Authors:** Jan E. Storm, Kimberly A. Mazor

**Affiliations:** New York State Department of Health, Center for Environmental Health, Troy, New York, E-mail: jes19@health.state.ny.us

In “Apartment Residents’ and Day Care Workers’ Exposures to Tetrachloroethylene and Deficits in Visual Contrast Sensitivity,” Schreiber et al. (2002) reported significantly lower visual contrast sensitivity (VCS) in apartment residents exposed to tetrachloroethylene (perchloroethylene, or perc) compared to unexposed “matched” control subjects. The authors stated that the VCS deficit may “represent a long-lasting, adverse alteration in neurobehavioral function” caused by chronic, environmental perc exposures, although they cautioned that methodologic limitations preclude definitive attribution of causation.

Residential data reported by Schreiber et al. (2002) were originally collected by the New York State Department of Health (NYSDOH) as a pilot project to support development of a larger study (NYSDOH, unpublished data). Residents exposed to perc included in the study were 13 adults from six households (20–72 years of age) and 4 children from three households (6–13 years of age) located in two buildings. Continued research by the NYSDOH and others (Farrar et al. 2001; NYSDOH 2004) suggests that confounding factors may influence VCS test performance of children in this and other studies. Consequently, we would like to update the findings of the residential study described by Schreiber et al. (2002).

In the analyses described by Schreiber et al. (2002), VCS of all perc-exposed adult and child residents and unexposed matched controls were compared using analysis of variance and SAS software (version 8.2; SAS Institute, Cary, NC). Matched pair, exposure (perc exposed, unexposed), and spatial frequency (cycles per degree) were independent variables; VCS was the dependent variable. The authors reported a significant effect of exposure on VCS (*F* = 19.38; df = 1,144; *p* < 0.001). Sample sizes were not sufficient to support statistical analysis of VCS stratified by age (i.e., child, adult); VCS data were available for only four children. However, review of individual VCS functions suggested that the significant VCS deficit was likely to be attributable to the four children in the exposed group. VCS functions of the exposed children were therefore carefully examined with respect to VCS functions for their matched controls and with respect to information about the children available from parental questionnaires.

Individual VCS functions for each exposed child were lower than his/her matched control (Figure 1A). Although perc exposure may have influenced VCS of these children, other factors could have contributed to their poor performance. For example, conditions such as developmental delay (DD) and attention deficit disorder (ADD) are known to be associated with decreased VCS (Farrar et al. 2001; Hudnell et al. 1996). One of the exposed children was characterized as having psychologist-diagnosed DD, and another exposed child was characterized as having physician-diagnosed ADD (Table 1). These two children performed poorly on the VCS but similar to unexposed children with similar diagnoses examined in a recently completed NYSDOH study (Figure 1B) (NYSDOH 2004). Also, another perc-exposed child was characterized as being forgetful at school, although not specifically as developmentally or learning disabled. (Questionnaires administered to residents of dry-cleaner buildings are part of NYSDOH records for the residential study; questionnaires were not completed for controls.) It is therefore possible that the perc exposure–VCS association reported by Schreiber et al. (2002) may have been confounded by the presence of these conditions.

In studies now being conducted by the NYSDOH and as reported by Scharre et al. (1990), 5- and 6-year-old children perform variably on the VCS test; sometimes they perform well, and sometimes they are inattentive and unable to perform. Two exposed children included in the residential study were 6 years of age. The matched control for one of these was 8 years of age, and the matched control for the other was the average of a 5-year-old and 7-year-old. Thus, although VCS was poor in perc-exposed child residents compared to others not exposed to perc, this may have been partly due to differences between groups in factors other than perc exposure (e.g., age).

In an exploratory analysis, VCS was evaluated only among adult participants in the residential study. When VCS of perc-exposed adult residents and unexposed adult control subjects were analyzed alone, excluding the four child pairs, a significant effect of perc exposure was not observed (*F* = 2.04; *df* = 1,108; *p* = 0.16). The sample size was small (*n* = 13) and consequently the statistical power was limited; however, the results suggest that VCS was not significantly decreased in perc-exposed adult residents.

Clearly, the possible effect of perc on VCS in adults, and especially in children, should continue to be explored. However, as illustrated here and discussed by Swinker and Burke (2002) and Hudnell and Shoemaker (2002), the possible influence of factors other than perc exposure on VCS should also be considered. These factors include age and the presence of learning disabilities or developmental delay in children, as illustrated here, as well as conditions such as diabetes, high blood pressure, glaucoma, and cataracts, in adults (Bodis-Wollner and Camisa 1980).

## Figures and Tables

**Figure 1:**
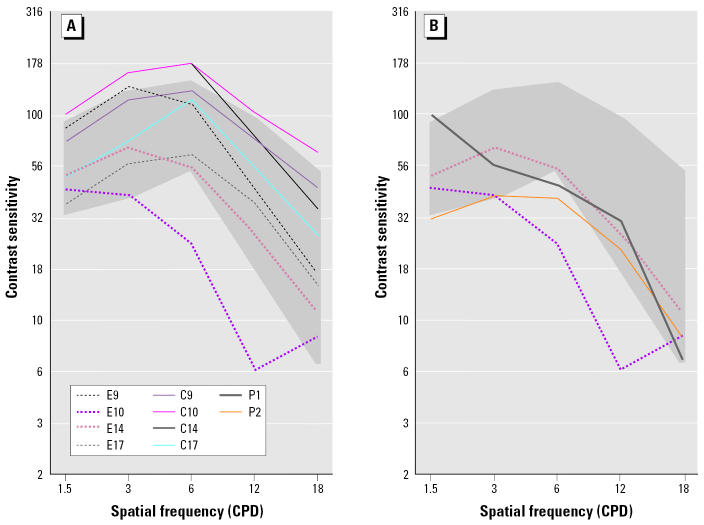
Individual VCS functions of children. (*A*) VCS functions of perc-exposed child residents (E9, E10, E14, E17) and matched controls (C9, C10, C14, C17) included by Schreiber et al. (2002) and the NYSDOH (2000). (*B*) Individual VCS functions of children characterized as having DD or ADD included by Schreiber et al. (2002; E10, E14) and examined in the NYSDOH study (NYSDOH 2004; P1, P2). The gray band reflects the normal adult range (90% confidence limits) reported for the Functional Acuity Contrast Test, F.A.C.T 101 (Stereo Optical Co., Inc., Chicago IL).

**Table 1:** Child residents and matched controls in the VCS studies.

Exposed			Matched control		
ID	Age	DD or ADD	ID	Age	DD or ADD
Children*^a^*					
E9	8	–	C9	9	–
E10	6	X	C10	8	–
E14	12	X	C14	12	–
E17	6	–	C17	5,7	–
Children*^b^*					
P1	8	X			
P2	10	X			

^a^Children shown in Figure 1A (NYSDOH, unpublished data; Schreiber et al. 2002).

^b^Children shown in Figure 1B; E10 and E14 from Schreiber et al. (2002) and P1 and P2 examined in the NYSDOH study (NYSDOH 2004).
